# Social networks, cultural pride, and historical loss among non-reservation American Indian/Alaska native emerging adults

**DOI:** 10.1186/s12889-025-25150-5

**Published:** 2025-12-03

**Authors:** David P. Kennedy, Ryan A. Brown, Elizabeth J. D’Amico, Daniel L. Dickerson, Carrie L. Johnson, Nipher Malika, Anthony Rodriguez, Virginia Arvizu-Sanchez

**Affiliations:** 1 RAND Corporation, 1776 Main St, Santa Monica, CA 90401 USA; 2 UCLA Integrated Substance Use and Addiction Programs (ISAP), Los Angeles, CA USA; 3 Sacred Path Indigenous Wellness Center, Los Angeles, CA USA

**Keywords:** egocentric networks, cultural pride, cultural pride, intergenerational trauma, cultural identity, traditional practices, personal relationships, emerging adults, non-reservation American Indian / Alaska Native, social networks

## Abstract

Generations of American Indian/Alaska Native (AI/AN) populations in the United States have endured disruptions to their community networks due to policies such as forced relocation and language prohibitions. Traumas resulting from these disruptions may have a ripple effect on subsequent generations’ mental, behavioral, and physical health. Evidence shows that cultural identity and participation in traditional practices promote resilience and protect health. Culturally-tailored health interventions customized for AI/AN people aim to address intergenerational trauma by emphasizing cultural strengths and building positive social connections. In this article, we explore the social network characteristics of participants of the first culturally-grounded health intervention for AI/AN emerging adults (18–25) living outside of reservations/Tribal lands. Participants (*N* = 469; 84.5% female) were recruited across the United States via social media and completed online egocentric network interviews prior to the start of intervention workshops. Participants’ networks were diverse in composition and structure. They were primarily composed of family and friends, were people they had regular contact with, were similar age, and provided participants with support. We examined associations between network characteristics, individual characteristics (age, gender, travel to reservations, speaking Tribal languages, etc.) and two dependent measures: (1) cultural pride and belongingness and (2) thoughts of historical loss. Multiple regression results show that having higher proportions of network members who discussed AI/AN identity with participants and having more network members who engage in traditional practices was positively associated with cultural pride and belongingness. Having denser egocentric networks was associated with lower cultural pride and belongingness. Higher proportions of network members having discussion of AI/AN identity with participants was also associated with more frequent thoughts of historical loss. Controlling for network factors, speaking a Tribal language at home with family was associated with higher cultural pride and belongingness. Also, respondents with mothers who obtained a college degree had less frequent thoughts of historical loss than those with mothers who did not have a college degree. We discuss implications for the development of culturally-grounded health interventions.

**Supplementary Information:**

The online version contains supplementary material available at 10.1186/s12889-025-25150-5.

## Introduction

Generations of AI/AN people in the United States (U.S.) have experienced social disruption due to forced relocation from traditional Tribal lands to designated U.S. cities, partly due to the Relocation Act of 1956 [1]. This relocation process contributed to the resettlement of a large number of AI/AN individuals to urban areas, where they experienced elevated rates of unemployment, homelessness, and disconnection from their community-based support networks [2, 3]. AI/AN people also experienced prohibitions against speaking their native languages, which is strongly connected to feelings of cultural connectedness [4, 5]. Forced relocation resulted in a disproportionate experience with trauma and post-traumatic stress [6], which has been linked to morbidity and mortality disparities for AI/AN populations [7–10]. The effects of colonization and forced relocation may have a ripple effect on subsequent generations’ mental, behavioral, and physical health [11–17]. Studies of AI/AN health disparities suggest that adverse social experiences may persist intergenerationally through a process called “embodiment” in which trauma and post-traumatic stress can have detrimental physiological effects that persist intergenerationally [18]. Repeated thoughts about these generational experiences can be psychologically distressing for Indigenous adolescents [5, 19, 20] and sexual minority adults [21], and are associated with suicide ideation [9, 10, 22, 23], behavioral addiction [24, 25], and depression [26–29].

In the face of these historical and ongoing challenges, AI/AN people have demonstrated tremendous resilience [30], highlighting need to investigate factors that provide protection [31–33]. Many AI/AN people who have spent most of their lives living outside reservation/Tribal areas maintain strong connections to their cultural and Tribal identities, other AI/AN people, and their Tribal lands [34, 35]. A growing body of evidence has linked strong cultural identity, which connects one to a sense of self and culture and empowers individuals to draw on cultural strengths when faced with challenges, to protection against emotional/behavioral health issues [36, 37] and promotion of resilience [30, 38–42]. AI/AN people who take part in traditional land-based subsistence and ceremonial activities report experiencing physical, spiritual, mental, emotional, and community benefits [43] and frequent visits to reservation/Tribal lands by non-reservation AI/AN emerging adults is associated with reduced physical pain [44].

Many recent interventions have been developed to improve the health of AI/AN populations by promoting positive concepts of AI/AN identity, encouraging engagement in traditional practices and increased community involvement [45–57], and improving social connections among AI/AN people [12, 58]. Health interventions have also been culturally tailored for AI/AN people living in urban areas [31, 59–62]. These programs focus on increasing overall well-being and healthy behaviors by addressing cultural identity, stigma, and community connections - as well as providing ways to promote health through engagement in traditional practices and culture – which is often protective and associated with decreased risk behavior [52–55, 59, 60, 63–65]. In addition to promoting cultural strengths, some programs also explicitly aim to reduce the negative effects of historical trauma for Indigenous populations [66–68].

Some researchers have called for expansion of AI/AN culturally-grounded interventions to include an emphasis on social networks and the role of social relationships in health [46]. An important aspect of many interventions that promote cultural strength and cultural pride is building a sense of social connectedness with other people who share the same AI/AN identity [12, 58]. Although ethnic identity is often assumed to be synonymous with demographic and/or biological characteristics, it is multi-faceted and strongly influenced by social factors [69]. Ethnic identity, similar to many other personal characteristics and behaviors, is shaped by existing social relationships [70] through two well established social network dynamics, network selection and influence [71]. Studies of adolescent development have found that peer networks are a key driver of the development of ethnic identity [72–74]. The social context of ethnic identity is especially important for interventions that address health disparities among AI/AN adolescents and emerging adults who do not live on reservations/Tribal lands. Non-reservation AI/AN adolescents and emerging adults may be socially isolated from other AI/AN peers as well as from family members and Tribal communities living in rural Tribal lands/reservations. Limited connections to people who share AI/AN identity coupled with extensive interactions and influences from non-AI/AN people in non-reservation areas is a potential barrier to promoting positive concepts of AI/AN identity, engagement in traditional practices, and increased involvement in AI/AN community activities for adolescents and emerging adults living in non-reservation areas.

This challenge is heightened for emerging adults, who are also developing new social roles and expanding their social networks beyond their families and school-based friendships. Non-reservation emerging adults may become more connected to their culture as they age and spend more time learning about their Tribal heritage, including the history of losses suffered by AI/AN people [75]. On the other hand, older non-reservation emerging adults may become more removed from feeling connected to their heritage as they transition into adulthood and become more independent from their families [76, 77]. The challenge is also complicated by other social factors, such as the gendered experience of social and cultural connection [78, 79] and socioeconomic status (SES) [44]. Those who come from families with economic resources may be able to afford involvement in cultural activities that require travel, such as visiting reservations or attending youth targeted cultural activities (e.g. the Gathering of Native Americans (GONA) [80]). On the other hand, higher SES may increase the social interaction of AI/AN emerging adults with higher SES people, which may make them feel more aligned with the majority ethnic culture in the U.S. due to the network effect of homophily [81].

One barrier to developing culturally-grounded health interventions for AI/AN emerging adults living in non-reservation areas is the lack of research on their social networks. To date, social network research on AI/AN emerging adults is sparse, despite decades of findings in other populations linking social factors with health [82–85]. Very little research with AI/AN communities and emerging adults has addressed the importance of social networks in supporting well-being. The studies that have addressed associations between AI/AN networks and health behaviors have used a variety of methods for measuring social networks. Some studies have indirectly measured social networks with proxy measures of scales about social ties instead of measuring specific network connections [84]. Several studies that have measured networks directly investigated the role of social networks among AI/AN teens living in Tribal areas [84, 86, 87] or measured one type of social relationship, such as students of the same school [83]. Understanding the strengths and diversity of non-reservation AI/AN emerging adults requires methods that capture information about a broad range of types of social relationships they may have, including strong and tightly connected ties (e.g. family members, AI/AN friends, etc.) as well as “weaker” and more diverse ties (e.g. school friends, work colleagues, mentors, neighbors, etc.). Capturing data about specific relationships among diverse types of contacts allows for investigations of different types of “social capital” available in their social networks [88–91]. For example, tightly connected social networks are high in social cohesion and support (“bonding” social capital) whereas loosely connected networks are helpful in providing a diversity of resources, information, and types of support (“bridging” social capital) [92–95]. Also, precise assessment of connections among network members enables assessment of the connectivity (“centrality”) of network members to assess the extent that these members are interconnected with the broader network and in a position to interact with and influence other network members [96].

### Current study

This study addresses these gaps by presenting empirical findings about the social networks of non-reservation AI/AN emerging adults. Findings can help inform development of culturally-grounded health interventions to address health disparities resulting from historical, inter-generational, and ongoing trauma and discrimination. Our team has conducted the largest study of social networks of non-reservation AI/AN emerging adults [31, 44, 97–100] using personal network (also referred to as “egocentric”) methods [101, 102]. We present results of analysis of the baseline survey with participants of a randomized controlled trial, TACUNA (Traditions and Connections for Urban Native Americans), which tests efficacy of a workshop-based, culturally-grounded substance use prevention intervention for non-reservation AI/AN emerging adults [103].

Our first aim is to describe the social networks of 469 non-reservation AI/AN emerging adults who were recruited for TACUNA. We examine the types of people in their network (e.g., friends, family, co-workers), age of social network members, geographic distance from network members, how frequently they interact with network members, and how many people in their personal networks provide support to participants. We also examine the proportion of network members who identify as AI/AN, engage in traditional practices, and discuss AI/AN identity with our survey participants. Finally, we analyze the structure of the participants’ networks, specifically how interconnected the members of these networks are to each other. We anticipate that AI/AN emerging adults living outside of reservations or Tribal lands will have many types of people in their lives, including a mixture of AI/AN and non-AI/AN connections.

Our second aim is to test for associations between social network characteristics and two key components of many culturally-grounded health interventions for AI/AN people: (1) cultural pride and belonging and (2) thoughts of historical loss. We examine these associations using regression models. We hypothesize that non-reservation AI/AN emerging adults who have more network members who report AI/AN identity, who engage in traditional practices, with whom they regularly discuss their AI/AN heritage, and who have experienced living on reservations/Tribal lands will have stronger feelings of connectivity to their cultural heritage and will also be more preoccupied with thoughts about the losses of culture and land experienced by generations of AI/AN people as a result of colonization. We also expect that structural characteristics of personal networks, such as overall network density and the extent that network members are connected to other network members who have AI/AN identity, will be associated with feelings of cultural connection. We hypothesize that densely connected personal networks consisting of many interconnected AI/AN people could provide a positive support and a social bonding context for positive experiences of AI/AN identity. However, because networks with high bonding capital also exhibit strong in-group solidarity and exclusion of out-group members [93], highly dense networks of mostly non-AI/AN people may be associated with lower cultural pride and belonging for non-reservation AI/AN emerging adults.

Finally, our third aim is to test for associations between individual characteristics of participants and cultural pride and belonging and thoughts of historical loss. The individual characteristics we examine include age, sex/gender minority identity, socio-economic status, if participants were born on a reservation/Tribal land, how much of their lives they have lived in non-reservation vs. reservation/Tribal lands, and if they speak a Tribal language. We hypothesize that demographic characteristics, such as age, gender, and socio-economic status, will be associated with these two outcomes. We also hypothesize that exposure to reservations/Tribal lands and speaking a traditional language at home with families will be significantly associated with a greater connection between AI/AN emerging adults and their cultural heritage. In contrast, we hypothesize that higher proportion of time spent living in urban areas will present a challenge to maintaining a strong cultural connection.

### Data and method

Participants (*N* = 469) were part of a randomized controlled trial, TACUNA, testing effects of two culturally grounded interventions on alcohol and other drug (AOD) use and cultural connectedness [103]. Data collection for the current study began in the early stages of the COVID-19 pandemic, December 2020, and continued through December 2023; therefore, recruitment occurred online via social media across the U.S., and participants completed surveys online. Participants who responded to an online advertisement were directed to an online screener to determine eligibility. Eligibility criteria included: (1) age 18 to 25, (2) living in an area in any state in the U.S. that is not on a rancheria or a reservation), (3) self-identification as AI/AN, and (4) English speaking. The parent trial is specifically focused on prevention of opioid use disorder and therefore focused on emerging adults who were not in need of treatment. Participants were screened for the absence of an opioid use disorder with the Rapid Opioid Dependence Screener [104]. Those who completed the screener and were eligible were contacted by staff from the RAND Survey Research Group, who informed them of their rights as participants; that their participation was voluntary; the study objectives; what they would be asked to do if they consent to participate; any potential costs, benefits, or risks of participation; the steps the project will take to keep their responses confidential; and the process of de-identifying data that the project is collecting. Those who consented to participate were then asked to complete an online survey and randomized to receive either one virtual workshop or three virtual workshops and a Wellness Circle [103]. Data for this paper originate from the baseline survey prior to participation in workshops. Participants received a $40 Amazon gift card upon survey completion. Procedures were approved by the RAND Human Subjects Protection Committee (HSPC) and by the project’s Urban Intertribal Native American Review Board, including procedures for obtaining informed consent.

### Measures

*Individual characteristics.* Respondents were asked a series of survey questions about their own demographic characteristics. We used the education level of their mothers (less than high school, high school, some college/AA degree, BA), as a proxy for SES [105], the educational level of their mothers (less than high school, high school, some college/AA degree, BA). Respondents also reported their age in years. In two separate questions, respondents reported the percent of their life they had spent in urban areas and percent of their life spent on reservations or Tribal lands (0–100%). They also reported on the frequency they traveled to reservation/Tribal lands in the past year (never, 1–30 days, or >30 days), if they were born on reservation/Tribal lands, and if they usually speak a Tribal language at home with families. To measure sexual gender minority identity broadly and to test for associations with cultural identity variables, respondents answered a series of questions to determine their sexual gender minority (SGM) identity. They reported their sex at birth, gender and transgender identification, sexual orientation, and gender(s) of past sexual partners. Participants could choose from a variety of answers to identify themselves demographically. For example, for what best describes their gender identity, participants could choose: (a) female, (b) male, (c) gender fluid, (d) something else, or (e) prefer not to say. We defined respondents as SGM individuals if they indicated any orientation other than “straight/heterosexual,” sex at birth being “something else” gender identity as “gender fluid” or “something else,” transgender identity, history of same-gender sex, or discordance between sex at birth and gender identity.

*Social Network Measures.* To capture a broad range of social contacts that span different social contexts and include both strongly and weakly tied network members, we measured respondents’ social networks with a personal network survey instrument [61, 101, 102]. This provided the raw data to produce measures of network composition (the types of people in the network) and network structure (the size and interconnections of this group of people). Personal network data are elicited using survey instruments that include one or more “name generators”, which prompt focal respondents (“egos”) to list network contacts (“alters”) based on their cognitions of their social environment [101, 102]. Egos are then asked a series of “name interpreters”, which prompt them to characterize each of these alters and their relationships with them. Rather than aiming to produce a full and objective enumeration of each ego’s entire social network, which could include hundreds of meaningful ties [106] and thousands of acquaintances [107], personal network studies capture responses about a more limited set of contacts that is large enough to capture diversity in social ties to meet research objectives [108], while limited enough to be reasonably assessed in an interview setting without overly burdening respondents [109, 110].

We asked respondents to name up to 15 people whom they talked with the most over the past three months (including in-person, phone, texting/emailing, etc.). We asked respondents what their relationship was with each alter (Was the alter a family member, friend, romantic partner, etc.?). Based on these responses, we calculated summary statistics for each respondent network. We generated a measure of proportions of alters in each relationship type for each respondent, including proportion “family”, “friend”, “romantic partner”, “co-worker”, “classmate”, “neighbor”, “teacher”, and “coach”. We also asked respondents to classify each alter into an age category relative to their own age and generated a measure of proportion of alters who were “older”, “around my age”, or “younger” age. Respondents rated how close they were to each alter with a series of questions addressing different dimensions of relationship strength. Respondents reported the distance each alter lived from them (same household, 0–5 miles, 5–15 miles, 15–50 miles, > 50 miles) and the frequency of contact they had with the alter (weekly, monthly, or less than monthly). Respondents indicated if they received different types of support from each network alter, including emotional support or encouragement, informational support (advice), or tangible support (money, transportation, food, or other things).

Respondents were also asked to report the AI/AN identity of each alter. Respondents were asked if alters identify as AI/AN (do they “think of themselves as American Indian/Alaska Native”, yes/no). For those whom the respondent indicated that they do identify as AI/AN, they were asked if the alter does or does not engage in AI/AN cultural/traditional activities and if the alter ever lived on a reservation or other Tribal land. For all alters, respondents indicated if they ever discussed their own AI/AN identity with the alter and how recently this discussion happened (within the past 3 months, between 3 and 12 months, or over 12 months ago).

To generate relationship data among the network alters named in each respondent’s personal network, we asked each respondent to evaluate if each unique pair of alters know each other. From these responses, we generated two types of measures of personal network structure that have been used to assess network cohesion and social capital: density [96, 111] and average degree [112]. Density is a ratio of the overall number of connections among alters (the number of alter-alter pairs who know each other, excluding the ego) to the maximum number of unique pairs of alters ((n*(*n*−1))/2 for a network the size of *n*). Density ranges from 0 (no connections) to 1 (everyone is connected). High density is an indication of “bonding” capital, whereas lower density networks that are not extremely disconnected have “bridging” capital [92]. To measure connectivity of individual network members, we calculate *degree* for each network member, which is defined by the number of other network members that each alter knows. To measure the average connectivity of AI/AN alters within a personal network, we calculate the average connectivity to AI/AN alters by summing the degree for each AI/AN alter and dividing by the egocentric network size. This measure is an assessment of the amount of connectivity alters in the network have with other AI/AN alters, which places them in a position to interact with the participants about AI/AN life experiences and culture.

*Dependent Variables.* We measured the connection between the respondent and their report of cultural pride and belonging and thoughts of historical loss. We assessed participants’ sense of cultural pride and belonging with the Multigroup Ethnic Identity Measure (MEIM) adapted for AI/AN heritage and used previously with a sample of AI/AN adolescents living outside of reservations/Tribal lands [113]. Respondents are asked the degree to which they agree with twelve statements such as, ‘I have a clear sense of my AI/AN identity and what it means to me’ on a scale from 1 = ‘strongly disagree’ to 5 = ‘strongly agree’ [114, 115]. We also assessed internalizations of the experiences of generational trauma for participants that can be psychologically distressing using the Historical Loss (HL) scale [5, 19, 20]. This measure was developed to measure the frequency with which Indigenous individuals contemplate losses to their culture, land, and people resulting from European colonization. Additional details about the MEIM and HL scales, including detail question and response prompt text are available in the Appendix.

### Analyses

To address our first aim, we produced summary measures of network composition and structure for each respondent network and analyzed these measures across the sample with summary descriptive measures, including mean, standard deviations, minimum, and maximum. To summarize composition measures at the personal network level, we counted the number of network members with a specific characteristic and then divided this count by the total number of alters named by the respondent to produce a proportion for each network measure. We also calculated summary measures of network structure for the full sample based on the individual measures of each personal network. Finally, we present summary measures (means, standard deviations for continuous variables and percentages for categorical measures) of the characteristics of the respondents.

To address our second and third aims, we constructed two multiple ordinary-least squares (OLS) regression models to test associations between network characteristics and individual participant characteristics with each of our dependent variables (cultural pride and belonging and historical loss). These multivariable models were constructed to test for associations between measures of networks and individual characteristics while controlling for other variables in the model. To identify which variables to include in the multivariable model, we first tested a series of bivariate models that tested for significant associations between each candidate independent variable and each outcome variable. Each variable with a *p-value* < 0.10 was included in a multivariable model predicting each dependent variable. As respondents were asked to name a set number of alters (15), only a small number of respondents deviated from this instruction. Therefore, we do not include the count of alters in the multivariable models. Instead, we include a dichotomous indicator of egocentric network size (= 15 vs. <15) as a control in the multivariable model to account for any bias that may result from smaller networks. Because measures of network proportions ranged from 0 to 1, these measures were converted to deciles (multiplied by 10) to aid in interpretation. All analyses were run using R 4.2.1. Regression analyses were run using the *glm* function of the R stats package.

## Results

### Descriptive results

The 469 participants in the sample were mostly female (84.9%) and the average age was 22.3 (Table 1). Approximately two-thirds of the sample’s mothers had some education above High School with 39.0% having achieved a Bachelor’s degree. Respondents averaged 73.8% of their lives in urban areas and 22.3% of their lives on reservations/Tribal lands, with only 15.8% having been born on a reservation or Tribal land. Most respondents (52.5%) spent between 1 and 30 days traveling to reservations/Tribal lands in the past year with 18.6% traveling more than 30 days and 29% not having traveled at all. Most respondents did not usually speak a Tribal language at home (79.3%). Based on the combination of responses to questions about self-reported gender and sexual orientation, 48.5% reported SGM identity. The overall MEIM scores averaged 4.12, which is above an “Agree” response (α = 0.88). The overall HL scale averaged 3.32, which represents an overall frequency of thoughts between “weekly” and “daily” (α = 0.95). The average for each item fell into the range between “weekly” and “daily”.

**Table 1:** TACUNA sample characteristics (*n* = 469)

Participant Characteristics	Mean(sd)	(%)
Age	22.3 (2.2)	
Sex at birth		
Male		14.5
Female		84.9
Intersex/Other		0.6
Mother’s education		
Less than high school		10.4
High school		22.2
Some college/AA		25.6
Bachelor’s degree		39.0
Don’t know		2.8
% of life spent in urban areas^a^	73.8 (30.9)	
% of life spent in reservations or Tribal lands^a^	22.3 (29.2)	
Days traveled to reservation/Tribal lands in past year		
0 days		29.0
1 to 30 days		52.5
31 or more days		18.6
Born on a reservation or tribal lands		
Yes		15.8
No		84.2
Usually speak tribal language at home with family		
Yes		20.7
No		79.3
Gender		
Man		13.4
Woman		74.4
Gender fluid		7.2
Something else		4.3
Prefer not to say		0.6
Sexual orientation		
Straight/heterosexual		47.1
Gay		2.3
Lesbian		4.5
Bisexual		30.5
Questioning		7.0
Asexual		2.3
Something else		4.7
Prefer not to say		1.5
Identify as SGM		48.5
Cultural Pride and Belonging (MEIM)	4.12 (0.66)	
Historical Loss Scale (HL)	3.32 (1.14)	

^a^Time spent in urban and reservation/Tribal areas were asked in two separate questions. Respondents did not always provide answers that totaled 100%

Table 2 summarizes network composition and structure. Most respondents (87%) provided 15 names at the name generator prompt. The overall average number of alters named was 14.2 and the smallest network included only 2 alters. On average, the egocentric networks had a density of 0.45 with some networks being completely disconnected (density = 0.00) and others having fully connected networks (density = 1.00). Networks were composed mostly of friends and family members; the average proportion of friends in the full sample of egocentric networks was 0.425 and the average proportion of family members was 0.350. Other types of network members named most frequently included co-workers (0.078), romantic partners (0.066), classmates (0.016), teachers (0.011), neighbors (0.005), and coaches (0.003). On average, respondents’ networks had a proportion of 0.583 alters whom they classified as “around my age”, 0.366 who were older, and 0.042 who were younger. Respondents’ networks had an average proportion of 0.568 alters they had contact with at least once a week, 0.265 once a month or more (but less than weekly), and 0.146 less than once per month. Respondents reported receiving emotional support (0.755) and information support (0.721) from most of the alters they named. On average, respondents reported receiving tangible support from almost half of the members of their personal networks (0.456). Fewer than half (0.433) of alters named in the personal networks of the respondents identified as AI/AN and around a quarter (0.261) engaged in traditional AI/AN activities. An average proportion of 0.250 of alters in respondents’ networks had ever lived on a reservation or Tribal lands. Respondents’ networks had an average proportion of 0.235 with whom respondents had never discussed their AI/AN identity. They discussed their AI/AN identity with around three-fourths of their networks, including around half (0.490) within the past 3 months, 0.169 within the past year (but not the past 3 months), and 0.068 over 12 months ago. On average, there were 2.79 connections to AI/AN alters across all respondent personal networks.

**Table 2:** Average egocentric characteristics (*n* = 469)

Variable Type	Social Network Variable	Mean (sd)	Range [min, max]
Network Structure			
	Egocentric Network Size	14.2 (2.48)	[2, 15]
	Density	0.45 (0.20)	[0.00, 1.00]
Network Composition (%)			
Relationship Type	Friend	0.425 (0.230)	[0.00, 1.00]
	Family	0.350 (0.225)	[0.00, 0.93]
	Co-worker	0.078 (0.129)	[0.00, 0.67]
	Romantic Partner	0.066 (0.069)	[0.00, 0.50]
	Classmate	0.016 (0.045)	[0.00, 0.27]
	Teacher	0.011 (0.035)	[0.00, 0.27]
	Neighbor	0.005 (0.027)	[0.00, 0.27]
	Coach	0.003 (0.002)	[0.00, 0.27]
Age	Same	0.583 (0.230)	[0.00, 1.00]
	Older	0.366 (0.216)	[0.00, 1.00]
	Younger	0.042 (0.077)	[0.00, 0.53]
Distance Live	Same household	0.117 (0.132)	[0.00, 1.00]
	Between 0 and 5 miles	0.176 (0.207)	[0.00, 1.00]
	Between 5 and 15 miles	0.191 (0.203)	[0.00, 1.00]
	Between 15 and 50 miles	0.130 (0.183)	[0.00, 1.00]
	Over 50 miles	0.339 (0.266)	[0.00, 1.00]
Frequency of Contact	Weekly	0.568 (0.232)	[0.00, 1.00]
	Monthly	0.265 (0.175)	[0.00, 0.87]
	Less than monthly	0.146 (0.166)	[0.00, 0.87]
Support	Receives emotional support	0.755 (0.259)	[0.00, 1.00]
	Receives tangible support	0.456 (0.298)	[0.00, 1.00]
	Receives information support	0.721 (0.280)	[0.00, 1.00]
AI/AN Identity	Identifies as AI/AN	0.433 (0.267)	[0.00, 1.00]
	Engages in AI/AN traditional practices	0.261 (0.233)	[0.00, 1.00]
	Lived on reservation or other Tribal land	0.250 (0.276)	[0.00, 1.00]
Discussed AI/AN Identity	Never	0.235 (0.288)	[0.00, 1.00]
	Within past 3 months	0.490 (0.350)	[0.00, 1.00]
	Between 3 and 12 months ago	0.169 (0.260)	[0.00, 1.00]
	Over 12 months ago	0.068 (0.178)	[0.00, 1.00]
Network Centrality	Average degree to AI/AN alters	2.79 (2.39)	[0, 12.7]

**Fig. 1:**
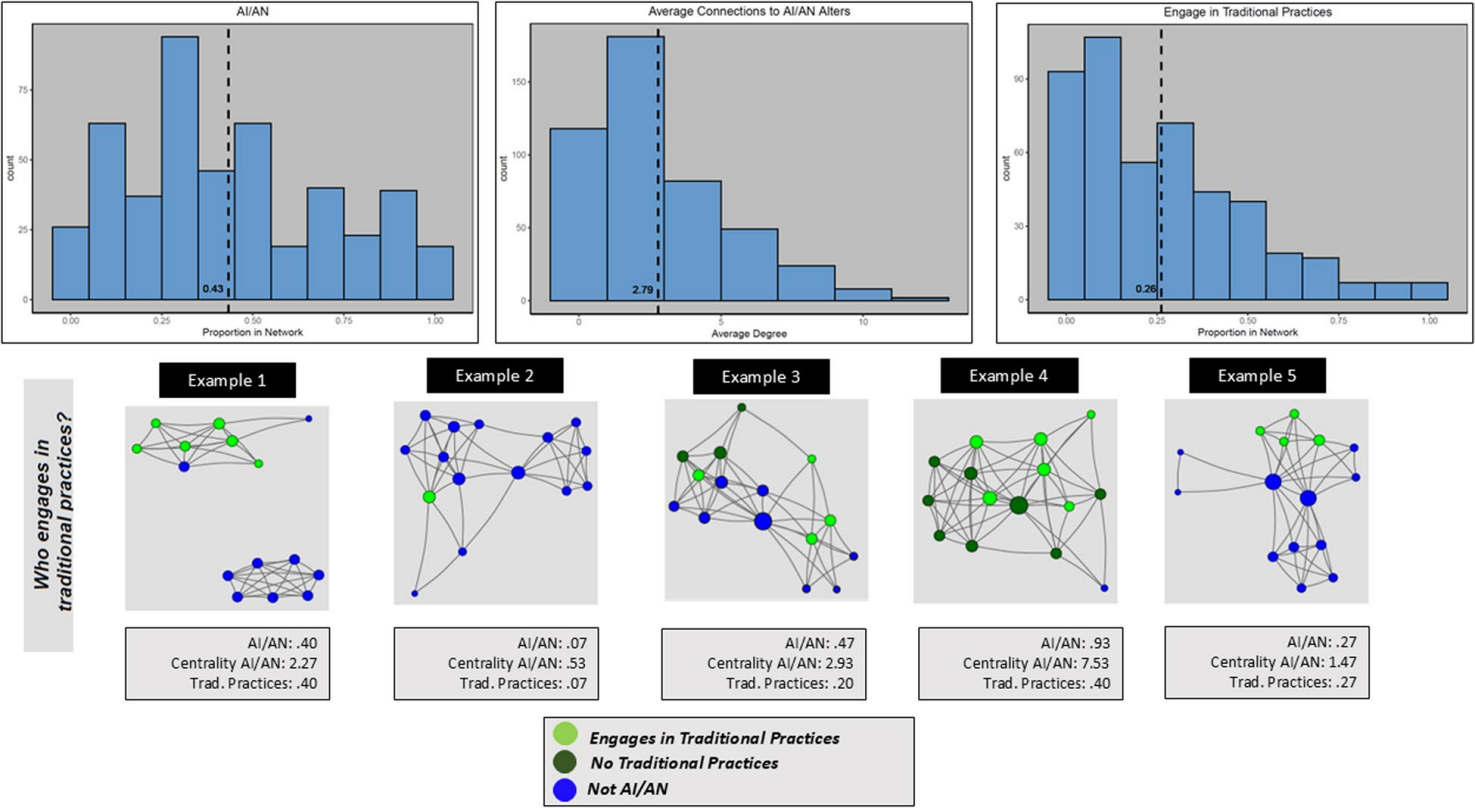
Figure 1. Distribution of AI/AN Discussion Partners and Example Personal Networks. Histograms showing distribution of three network measures: (left) Proportion of alters who identify as AI/AN; (center) Average connections (degree) to AI/AN alters; (right) Proportion of alters who engage in traditional practices. The figure also presents five example respondent egocentric networks illustrating the three measures. Each of the diagrams illustrate characteristics of alters with colored circles (“nodes”) and connections between alters as lines (“edges”). The nodes are placed on the plot with the R package “igraph” using the Fruchterman–Reingold spring embedding layout based on edges defined as alters who know each other. The light green nodes depict alters who identify as AI/AN and engage in traditional practices. Dark green nodes identify as AI/AN but do not engage in traditional practices and blue nodes do not identify as AI/AN. Node size represents the number of connections the node has to other nodes in the graph (“degree centrality”). Each example network diagram is illustrated with three measures: % alters who identify as AI/AN, average centrality of AI/AN alters, and % of alters who engage in traditional practices

**Fig. 2:**
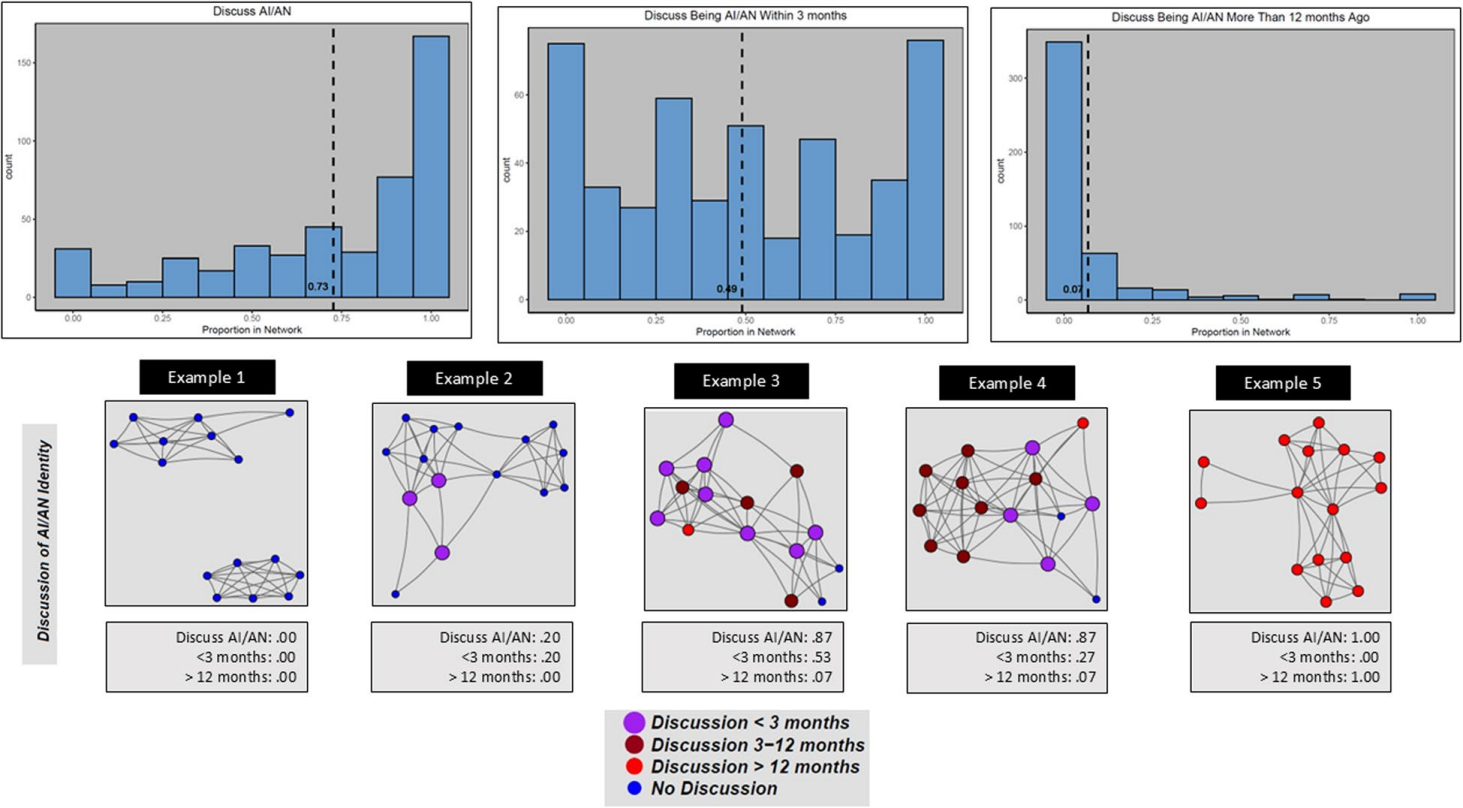
Figure 2. Distribution of AI/AN Status and Example Personal Networks. Histograms showing distribution of three network measures: (left) Proportion of alters with whom the respondent has discussed being AI/AN; (center) Proportion of alters with whom the respondent has discussed being AI/AN within the past 3 months; (right) Proportion of alters with whom the respondent has discussed being AI/AN more than 12 months ago. The figure also presents five example respondent egocentric networks illustrating the three measures. Each of the diagrams illustrate characteristics of alters with colored circles (“nodes”) and connections between alters as lines (“edges”). The nodes are placed on the plot with the R package “igraph” using the Fruchterman–Reingold spring embedding layout based on edges defined as alters who know each other. Node size and color represent how recent the respondent has discussed being AI/AN with the alter with the largest nodes representing most recent (light purple nodes) and the smallest nodes representing those with whom the respondent has never discussed being AI/AN (small blue nodes)

Figure 1 illustrates the distribution of network characteristics across the sample, with histograms for proportion of alters identifying as AI/AN (left), average centrality of AI/AN alters (center), and the proportion of alters who engage in traditional practices (right). Figures 1 and 2 also provide a visual illustration of five respondent networks. The figures highlight three network measures (proportion of AI/AN alters, proportion of alters who engage in traditional practices, and the average degree centrality of AI/AN alters). Figure 1 demonstrates that there is a mostly even distribution of proportion of AI/AN alters with a spike around 0.30 and an average proportion above 0.40. The example networks 1 and 5 are closest examples of mean. The distribution of average degree of AI/AN alters is skewed to the right, with an average near 3 connections. Most of the networks have an average AI/AN degree of fewer than 5, with a small number of networks with higher average AI/AN alter interconnectivity. Example networks 1 and 3 are the closest to the sample mean. The distribution of the proportion of alters who engage in traditional practices is skewed to the right with a mean below 0.30. There is a small number of networks where nearly everyone engages in traditional practices. Example 5 is the network a proportion closest to overall sample mean proportion of alters engaging in traditional practices.

In Fig. 2, proportions of discussions with network members about AI/AN identity is skewed to the left. The largest number of respondents discussed AI/AN with everyone or nearly everyone in their networks. Example 5 represents a network in which all alters discussed being AI/AN with the respondent. The proportion of discussions with alters recently (within 3 months) is more evenly distributed with an average of roughly 0.50. Example 3 is the example network that most closely demonstrates the mean. The distribution of the proportion of discussions with alters longer than one year ago is skewed to the right with mostly no recent discussions or low proportions of less recent discussions with some having close to all network members fitting into this category. Example 5 is an example of a network in which the respondent reported having had discussions more than one year ago with each alter.

### Bivariate results

Table 3 presents results of bivariate tests of association between each of the network and individual measures and the MEIM/HL scale averages. Each row presents results of two different bivariate tests, one for each outcome variable. The table provides model estimates, 95% confidence intervals, and *p*-values. Results indicate that the following individual characteristics were significantly associated with MEIM scores: mother’s education, amount of time lived in urban areas, amount of time lived in reservations/Tribal lands, days visiting Tribal lands, being born on a reservation, and speaking a Tribal language. Several network variables were also significantly associated with MEIM, including proportion of alters who were AI/AN, proportion who engage in traditional activities, proportion of alters whom the respondent discussed AI/AN in the past 3 months and over 1 year ago, and proportion of alters who have lived on reservations. Overall network density and the average number of connections to AI/AN alters were significantly associated with MEIM scores.

**Table 3:** Bivariate OLS regression models predicting MEIM and HL (*n* = 469)

	MEIM			Historical Loss		
Variable	Est	95% CI	*p*-val	Est	95% CI	*p*-val
Age	0.005	(−0.020,0.031)	0.67	0.051	(0.008,0.095)	0.02
Mother education – BA (vs. less than BA)	0.131	(0.017,0.245)	0.03	− 0.284	(−0.481, − 0.087)	< 0.01
Female gender	− 0.006	(−0.154,0.142)	0.94	0.043	(−0.213,0.300)	0.74
% of life lived in urban area	− 0.002	(−0.004,−0.001)	0.01	0.000	(−0.003,0.003)	0.78
% of life lived in reservations or Tribal lands	0.003	(0.001,0.005)	< 0.01	0.000	(−0.003,0.003)	0.88
Days visiting reservations or Tribal lands	0.241	(0.162,0.320)	< 0.01	0.104	(−0.038,0.246)	0.15
Born on reservation or Tribal lands	0.19	(0.040,0.339)	0.01	− 0.038	(−0.300,0.223)	0.77
Usually speak Tribal language at home with family	0.322	(0.190,0.455)	< 0.01	0.193	(−0.041,0.427)	0.11
Sexual/Gender Minority	0.005	(−0.106,0.116)	0.93	0.193	(0.050,0.432)	0.01
Proportion Alters who think of themselves as AI	0.069	(0.049,0.088)	< 0.01	0.034	(−0.002,0.070)	0.07
Proportion alters who engage in traditional activities	0.102	(0.080,0.125)	< 0.01	0.065	(0.023,0.107)	< 0.01
Proportion of alters respondent has discussed being AI/AN < 3 months	0.05	(0.034,0.066)	< 0.01	0.112	(0.085,0.139)	< 0.01
Proportion of alters respondent has discussed being AI/AN 3–12 months	0.012	(−0.010,0.035)	0.28	− 0.039	(−0.078, − 0.001)	0.05
Proportion of alters respondent has discussed being AI/AN >12 months	− 0.048	(−0.080,−0.015)	< 0.01	− 0.084	(−0.140, − 0.027)	< 0.01
Proportion alters who lived on reservation	0.059	(0.038,0.079)	< 0.01	0.019	(−0.018,0.056)	0.31
Density of Egocentric Network	− 0.399	(−0.688,−0.110)	0.01	− 0.489	(−0.991,0.014)	0.06
Average # of Connections to AI Alters	0.162	(0.033,0.290)	0.01	0.166	(−0.058,0.389)	0.15

For HL scores, age, mother’s education, and SGM status were the individual characteristic measures significantly associated with HL scale scores. Most network variables were significantly associated with HL scores, including proportion of alters who were AI/AN, proportion who engage in traditional activities, and proportion of alters whom the respondent discussed AI/AN in the past 3 months, 3–12 months, and over 1 year ago. The density of egocentric networks was also significantly associated with higher scores on the HL scale. The average number of connections to AI/AN alters was not significantly associated with the HL scale.

### Multivariable results

Table 4 provides estimates, 95% CIs, and *p*-values for each full multivariable model predicting MEIM and HL scores. Controlling for network factors, respondents who spoke a Tribal language at home/with their families scored 0.233 higher MEIM scores on average relative to those who did not speak a Tribal language. Controlling for individual characteristics, several network measures were associated with MEIM scores. Respondents with higher proportions of alters who engaged in traditional activities had significantly higher MEIM scores on average. For each 10% increase in proportion of alters who engaged in traditional activities there was a predicted 0.071 increase in the MEIM score. Discussing AI/AN identity was also significantly associated with MEIM scores. An increase in 10% of alters with whom the respondent spoke about AI/AN identity increased MEIM scores by 0.039. Egocentric network density was significantly associated with lower MEIM scores; each 10 point increase in density was associated with a 0.60 lower MEIM score on average. Other network measures were not significantly related to MEIM score.

**Table 4:** Multivariable OLS regression models predicting MEIM and HL (*n* = 469)

	MEIM			Historical Loss		
Independent Variables^*^	**Est**	**95% CI**	**p-val**	**Est**	**95% CI**	*p*-val
Age	--	--	--	0.015	(−0.028,0.058)	0.49
Mother education – BA (vs. less than BA)	0.076	(−0.03,0.18)	0.17	− 0.390	(−0.586,−0.194)	< 0.001
% of life lived in urban area	− 0.003	(−0.01,0.00)	0.05			
% of life lived in reservations or Tribal lands	− 0.002	(−0.01,0.00)	0.41			
Days visiting reservations or Tribal lands	0.056	(−0.03,0.14)	0.21			
Born on reservation or Tribal lands	− 0.013	(−0.17,0.14)	0.88			
Usually speak Tribal language at home with family	0.233	(0.09,0.37)	< 0.001			
Sexual/Gender Minority				0.152	(−0.039,0.344)	0.12
Proportion Alters who think of themselves as AI	0.005	(−0.04,0.05)	0.84	0.016	(−0.035,0.066)	0.55
Proportion alters who engage in traditional activities	0.071	(0.04,0.10)	< 0.001	0.029	(−0.031,0.088)	0.35
Proportion of alters respondent has discussed being AI/AN < 3 months	0.039	(0.02,0.05)	< 0.001	0.124	(0.090,0.157)	< 0.001
Proportion of alters respondent has discussed being AI/AN 3–12 months				0.031	(−0.011,0.074)	0.15
Proportion of alters respondent has discussed being AI/AN > 12 months	− 0.114	(−0.42,0.19)	0.47	0.000	(−0.057,0.056)	0.99
Proportion alters who ever lived on reservation	− 0.019	(−0.05,0.01)	0.27			
Density of Egocentric Network	− 0.600	(−0.98,−0.22)	< 0.001	− 0.292	(−0.788,0.204)	0.25
Average # of Connections to AI/AN Alters	− 0.031	(−0.02,0.08)	0.26			

^*^Controlling for egocentric network size = 15 vs. <15

For the model predicting HL scores, the only individual measure significantly associated with HL, controlling for other factors, was mother’s education. The HL scores of those who had mothers who had achieved at least a BA degree were 0.390 lower, on average, than respondents with mothers with less education. Controlling for individual factors, respondents with higher proportions of alters with whom the respondent spoke to about being AI/AN recently tended to have higher HL scores, on average. For each 10% increase in alters with whom the respondent spoke to about being AI/AN within the past 3 months, the HL score increased by 0.124. Age and sexual/gender minority status was not significantly associated with HL scores.

## Discussion and conclusions

The current study is the first to present a detailed analysis of the social networks of non-reservation AI/AN emerging adults and provides empirical insights about the relationship between their social networks and their feelings of cultural pride and belonging as well as their thoughts of historical loss. These insights can inform the continued development of culturally-grounded health interventions for AI/AN populations. Understanding social networks among non-reservation AI/AN emerging adults may be important to inform ways to increase protective factors for this population. The continued health disparities experienced by AI/AN people in the U.S. indicate that existing evidence-based treatments are not addressing the needs of this population adequately [55]. Recent calls for innovative approaches to developing health interventions tailored for AI/AN populations suggest addressing social network factors that may be unique to AI/AN people [46, 86, 87].

Because of the lack of research on the social networks of non-reservation AI/AN emerging adults, we first aimed to describe their networks. Findings suggest that AI/AN emerging adults living in non-reservation areas have, on average, sources of both bonding and bridging capital. The descriptive statistics of their networks indicate that the study sample’s networks were composed of alters with many types of characteristics commonly associated with bonding capital [88–91], such as friends, family, similar age, and people they saw at least weekly. The average network also included many people from whom respondents reported receiving different types of support, including close to half from whom they received something tangible, such as money, transportation, food, etc. In addition, there were also indications of diversity of types of network members and network connectivity. For example, on average, the largest proportion of network members (around one-third) lived more than 50 miles away from respondents. Although respondents likely maintained contact with these people through electronic communication, physical proximity is a well-established component of strong ties, suggesting that geography may be a barrier to maintaining ties with these network members and may contribute to social fragmentation in respondents’ networks [81, 116].

The pattern of connections respondents reported having with other AI/AN people also indicates compositional and structural diversity. Respondent networks were less densely connected than a study of personal networks of Alaska Native adolescents, which found an average density of 0.98 [87], indicating more weakly tied alters for this non-reservation sample. Also, on average, fewer than half of participants’ networks were composed of other people with AI/AN identity and only a around a quarter of network members engaged in traditional AI/AN practices. This suggests that many of these AI/AN emerging adults are balancing different social worlds composed of AI/AN and non-AI/AN people as well as balancing connections to AI/AN people with differences in how strongly they are connected to AI/AN culture. AI/AN alters appear to be less structurally central than non-AI/AN alters because their average degree (2.79) is lower than the overall average degree across all alters in the full sample (5.93). Therefore, fewer than 3 people, on average, were connected to other AI/AN people in the respondents’ network. This may present a barrier for respondents who seek network members to interact with about how their AI/AN identity impacts their lives. It is possible that the non-AI-AN alters respondents named did not know any other AI/AN people personally. Respondents did report having discussed their AI/AN identity with roughly two-thirds of their networks, with close to one-half of these discussions happening in the most recent 3 months. The baseline survey did not capture information about what type of discussions respondents had with network members. It is possible that these conversations included discussions with other AI/AN network contacts about their shared cultural background while others may have involved discussions where respondents explained AI/AN culture to their non AI/AN network contacts, indicating high “brokerage”, which can provide benefits to those in networks with high “bridging” [91].

The second aim of this study was to test for association between these network characteristics and feelings of pride and belongingness for AI/AN emerging adults living in non-reservation areas. As anticipated, there was a significant association between measures on these constructs and how connected participants were with other AI/AN people in their social networks. Although we did not find an association between cultural pride and the overall proportion of alters who identified as AI/AN, controlling for individual characteristics and other network measures, stronger feelings of cultural pride and belonging were associated with higher amounts of network connections with AI/AN people who engaged in traditional practices. This finding is consistent with research related to substance use [97, 99] whereby engaging in traditional practices among AI/AN network members appears to be an important characteristic that is associated with positive outcomes for non-reservation AI/AN emerging adults. Cultural pride and belonging was also significantly associated with having larger proportions of network members with whom the respondent spoke to about AI/AN identity recently. Although we cannot characterize what respondents discussed when they had these conversations, it appears that having these discussions is associated with, and perhaps enhanced, cultural pride. On the other hand, respondents with higher cultural pride may have been more comfortable engaging in these discussions. Future studies with longitudinal assessments of network characteristics and cultural pride can provide evidence to test these alterative explanations. Finally, we found that density was negatively associated with cultural pride and belonging. It is possible that because AI/AN alters tended to be less central to the respondents’ networks than non-AI/AN networks, densely connected networks of mostly non-AI/AN people worked against positive feelings of AI/AN cultural pride.

Tests of association between network characteristics and thoughts of historical loss showed that respondents who had higher proportions of alters in their network with whom they engaged in discussions of being AI/AN in the past 3 months had significantly higher scores on the HL scale. It is not surprising that respondents who have frequent conversations about AI/AN identity with their social networks would have more thoughts of historically traumatic events that have impacted AI/AN people. Because thoughts of historical loss are associated with depressive symptoms among AI/AN adolescents [27], interventions that promote greater social connections and conversations between AI/AN people may increase recurring thoughts of historical loss, which could indirectly lead to an increase in depressive symptoms. However, other Tribally led, culturally-tailored health interventions that provide education about AI/AN historical trauma have found only short-term increases in thoughts of historical loss among program participants [117], and, by six-month post-test, thoughts of historical loss were no longer significantly increased. Studies have highlighted that interventions must also promote social connections and discussions with network members about AI/AN identity to help reduce negative health outcomes due to increased thoughts of historical loss [5, 66, 117–120]. Thus, programs that include discussions of historical trauma should include resources to support participants that may experience heightened mental health challenges as a result of increased thoughts of historical loss.

The final aim of the study was to test for associations between individual characteristics and cultural pride and belonging and historical loss scores, above and beyond the characteristics of their networks. Although several individual characteristics were significantly associated with cultural pride and belonging in tests of bivariate associations, many of these associations were not significant when included in a multivariable model that controlled for network characteristics. We did find that respondents who usually spoke a Tribal language at home with their families had significantly higher cultural pride and belonging scores, controlling for network factors. This is consistent with research that argues for the importance of speaking native languages in reinforcing feelings of cultural pride for AI/AN people [4, 5]. Speaking a Tribal language with family may protect emerging adults from a feeling of disconnection from their culture despite not living in a Tribal area and not having many AI/AN connections in their networks. We hypothesized that the amount of time respondents spent in either urban or reservation areas would be associated with cultural pride. Many respondents split their time between living in urban and reservation areas and reported spending time traveling back and forth between the two areas, similar to other studies of urban AI/AN people [34, 35]. Some studies of traditional land-based health interventions for urban AI/AN adults have found beneficial effects on urban AI/AN people who spend time in Tribal lands engaging in traditional, land-based subsistence activities [43]. Another study of the TACUNA baseline data found that vising Tribal lands was associated with lower pain experience, compared to those who did not visit at all [44]. Our bivariate model did find that higher percentage of time spent in urban areas was associated with lower cultural pride and belongingness. However, this association did not reach significance at the 95% confidence level in the multivariable model, when controlling for other measures, including network measures. It is possible that benefits to non-reservation AI/AN people visiting reservation/Tribal areas primarily operate through social contact with people who identify as AI/AN and engage in traditional practices.

The only individual characteristic significantly associated with frequency of thoughts of historical loss was mother’s education. Those with mothers who have not obtained a college degree, relative to those with mothers who obtained a BA or more, had more frequent thoughts of historical loss. One interpretation of this finding is that, because mother’s education is a proxy measure of higher SES [105], those with higher economic resources and opportunities may be less likely to have frequent thoughts about the losses experienced by their education. Another possibility is that having a mother with more education raises the likelihood that their children will receive cultural educational opportunities and opportunities to engage in traditional practices, which may help adolescents and emerging adults cope with the traumatic history of their ancestors. AI/AN families that live outside of reservation/Tribal areas often have challenges providing their children with opportunities to experience their cultural traditions [59]. Therefore, those with greater economic resources may be able to afford expenses associated with visiting Tribal lands [44] and/or participating in events that provided targeted cultural activities, such as GONA [80]. We hypothesized that higher SES may increase the social interaction of AI/AN emerging adults with higher SES people, which may make them feel more aligned with the majority ethnic culture in the U.S. due to the network effect of homophily [81]. This may also explain the negative association with the historical loss scale. However, we did not find SES to be associated with cultural pride and belonging, which contradicts this hypothesis.

Although this study has many strengths and provides empirical evidence lacking in the literature on the social networks of AI/AN emerging adults living in non-reservation areas, there are some limitations. First, the study is cross-sectional. Therefore, our findings cannot provide insights into causation and we are unable to determine if social network factors lead to cultural pride/belonging or thoughts of historical loss or if the network characteristics we observed are a result of characteristics of the respondents. Any interpretation of our findings using language that suggests causation is based only on speculation about possible implications of the findings. Another limitation is that respondents for this study were recruited for an ongoing RCT [103] and consented to participate in a series of intervention workshops addressing substance use. Although the RCT design, including participant randomization into two study arms and repeated assessments of individual and network characteristics over time, is a strength of the TACUNA project, there is likely some self-selection bias in the baseline sample. Another limitation of this study is that our sample is mostly female and includes a high proportion of sexual and gender diverse respondents. Further studies conducted among larger samples may offer an opportunity to further our understanding of the role of social networks and cultural identity on non-reservation AI/AN emerging adults; thus it is not representative. Further, although we controlled for other demographic characteristics in tests of associations with dependent variables and did not find any direct associations between them, it is possible that there are unknown biases that affect our findings.

Finally, the network data are generated through a personal network interview, which is based on proxy reports about other people rather than objective measures. This limitation is mitigated by the objectives of the study, which were to directly measure the perceived social environment of AI/AN emerging adults. Also, several studies of the accuracy of ego perceptions of alter characteristics compared to alter self-reports have found that they are often accurate when assessing observable characteristics [121, 122]. Another challenge to interpretation of our findings through comparison to other studies of personal networks, such as other studies of AI/AN people or other ethnic minority populations of emerging adults, is the variety of design options in personal network data collection instruments, such as quantity and types of name generators, types of name interpreters, number of alters to elicit, and whether to include assessments of ties among alters [101, 102, 108]. Although this flexibility is a strength, enabling the capture of data about social experiences meaningfully customized for specific populations, it also results in a proliferation of different types of designs and little consensus about the best methods for capturing personal network data [108].

A notable strength of capturing personal network data is the ability to provide participants in behavioral health interventions with immediate visual feedback about the answers they provided during an interview, which they often find enjoyable. The flexibility of personal network instruments enables adaptations of personal network feedback for different populations and behavioral health interventions targeting different outcomes [61, 123, 124]. However, it is currently unknown what impact different design choices have on the use of personal network visualizations in interventions. Future research comparing different approaches to capturing and visualizing personal network data would be useful to inform strategies for integrating personal networks into behavioral health interventions.

In conclusion, our findings provide empirical data about social network characteristics of AI/AN emerging adults living in non-reservation areas that have been lacking in the literature. This information is critical for developing culturally-grounded health interventions for this population to increase protective factors and help decrease the impact of inter-generational historical trauma. Findings suggest discussing social networks may be beneficial in programs that seek to improve health outcomes for non-reservation AI/AN emerging adults through promotion of cultural strengths, pride, and belongingness while also recognizing the traumatic history of AI/AN people. Although this study focuses on AI/AN people living in the United States, the findings are relevant for other displaced and historically traumatized populations around the world, such as international refugees or ethnic minority populations that are experiencing health disparities due to inter-generational trauma.

## Supplementary Information

Supplementary Material 1.

## Data Availability

The data underlying this article will be shared in a repository that is currently in progress. Contact the first author, Dr. David Kennedy, or the PIs of the project grants, Dr. Elizabeth J. D’Amico and Dr. Daniel L. Dickerson, with any queries.
