# Supplementary material for: Social networks, cultural pride, and historical loss among non-reservation American Indian/Alaska native emerging adults
Source: BMC Public Health. 2025 Dec 3;26:103. doi: 10.1186/s12889-025-25150-5 (PMC12781289; doi:10.1186/s12889-025-25150-5)
Supplement: Supplementary file 1 — Supplementary Material 1. [file 12889_2025_25150_MOESM1_ESM.docx]

Appendix

Table A1. Average ratings of Cultural Pride and Belonging (MEIM). Prompt: “The next questions ask about culture. and respond to each item using the following scale: strongly disagree, somewhat disagree, in the middle, somewhat agree, strongly agree.”^a^

| Item Text | Mean (sd) |
| --- | --- |
| I have spent time trying to find out more about my AI/AN identity | 4.42 (0.89) |
| I am active in groups that include mostly members of my AI/AN group | 3.29 (1.31) |
| I have a clear sense of my AI/AN identity and what it means for me | 3.92 (1.07) |
| I think a lot about how my life will be affected by my AI/AN identity | 4.06 (1.01) |
| I am happy that I am a member of the AI/AN tribal group I belong to | 4.60 (0.72) |
| I have a strong sense of belonging to my AI/AN tribal group | 3.84 (1.11) |
| I understand pretty well what my AI/AN identity means to me | 4.06 (1.00) |
| In order to learn more about my AI/AN identity, I have often talked to other people | 4.09 (1.01) |
| I have a lot of pride in my AI/AN identity | 4.65 (0.67) |
| I participate in cultural practices of my own AI/AN tribal group | 3.82 (1.15) |
| I feel a strong attachment towards my AI/AN tribal group | 4.26 (0.94) |
| I feel good about my cultural background | 4.42 (0.84) |
| Overall Scale Average: | 4.12 (0.66) |

^a^5-point scale from Response of “Strongly Disagree” = 1 to “Strongly Agree” = 5

Table A2. Average ratings of Historical Loss scale (HL). Prompt: “Our people have experienced many losses since we came into contact with Europeans (Whites). How often do you think of these things?” ^a^

| Item Text | Mean (sd) |
| --- | --- |
| The loss of our land | 3.37 (1.29) |
| The loss of our language | 3.48 (1.25) |
| Losing our traditional spiritual ways | 3.40 (1.31) |
| The loss of our family ties because of boarding/residential schools | 3.06 (1.49) |
| The loss of families from the reservation/reserve/village to government relocation | 3.03 (1.49) |
| The loss of self-respect from poor treatment by government officials | 3.29 (1.41) |
| The loss of trust in whites from broken treaties | 3.36 (1.41) |
| Losing our culture | 3.67 (1.28) |
| The losses from the effects of alcoholism on our people | 3.36 (1.39) |
| Loss of our people through early death | 3.18 (1.47) |
| Overall Scale Average: | 3.32 (1.14) |

^a^6-point scale “Never” = 0, “Several Times a Day” = 5
